# Large scale rigidity-based flexibility analysis of biomolecules

**DOI:** 10.1063/1.4942414

**Published:** 2016-02-18

**Authors:** Ileana Streinu

**Affiliations:** Department of Computer Science, Smith College, Northampton, Massachusetts 01063, USA

## Abstract

KINematics And RIgidity (KINARI) is an on-going project for *in silico* flexibility analysis of proteins. The new version of the software, *Kinari-2*, extends the functionality of our free web server KinariWeb, incorporates advanced web technologies, emphasizes the reproducibility of its experiments, and makes substantially improved tools available to the user. It is designed specifically for *large scale experiments*, in particular, for (a) very large molecules, including bioassemblies with high degree of symmetry such as viruses and crystals, (b) large collections of related biomolecules, such as those obtained through simulated dilutions, mutations, or conformational changes from various types of dynamics simulations, and (c) is intended to work as seemlessly as possible on the large, idiosyncratic, publicly available repository of biomolecules, the Protein Data Bank. We describe the system design, along with the main data processing, computational, mathematical, and validation challenges underlying this phase of the KINARI project.

## I. INTRODUCTION

Protein flexibility, especially the study of *large conformational changes*, is intimately related to protein function, yet its study poses enormous challenges. Current experimental methods are expensive and need further development to generate precise, high resolution dynamical data. A variety of computational methods and software implementations have been developed for studying the motions of biological molecules.

### A. Modeling protein flexibility and motion

Physics-based *molecular dynamics simulations* compute time-based trajectories of all atoms. Computationally, they are extremely intensive and slow, requiring access to large computer clusters or specialized hardware to examine a single structure on biological significant time scales. In spite of their success with *fast* protein motions,^25^ there is no solution in sight for *slow* functional motions.

*Coarse-grained simulation models* offer a compromise, sacrificing detailed, full atom information to efficiency yet hoping that accuracy is not lost in the process. Such methods group the molecule's atoms into *clusters* according to a variety of criteria, either chemical or distance-based, and treat each cluster through some representative. For example, *C_α_* atoms provide a coarse-grained representation for protein residues, but other residue-specific information may be lost if not incorporated by other means in the model. Atom interactions are usually approximated to shorter range versions (up to a cutoff distance). Popular coarse-grained approaches include the Gaussian Network Model (GNM) and its many variations. They view the interactions between protein *residues* as elastic springs, subject to Hooke's Law and thus inducing oscillatory motions. Normal modes are computed from an associated matrix and used to group the residues into *domains* and to infer large scale displacements among them. There is a vast literature on the subject, servers and third-party software are available,^37^ and studies^28^ have provided evidence of useful functional information that can be gathered in this manner. GNM has been shown to have good agreement with experimental crystallographic B-factors, which provide a measure of uncertainty (due to both experimental artifacts and motions within the molecule) for atomic positions in crystal structures solved with X-ray crystallography.^26^ GNM calculations have also been used to compute clusters of protein residues (GNM-based domains), which have been compared to the structural domains assigned by classification schemes (SCOP, CATH) and experimental crystallographers.^28^

### B. Rigidity analysis

In another category are computational methods that do not directly predict molecular motions; rather, they provide information on which groups of atoms in a molecule are *likely to move together* as rigid clusters. Such methods build a decomposition of a molecule into *rigid clusters* of atoms connected to form *flexible* networks. This gives a starting point for predicting possible motions and conformations of a molecule.

One such method is RigidFinder,^1^ which compares multiple X-ray structures or NMR (nucleic magnetic resonance) models of the same molecule. It interprets closely similar (up to trivial rigid transformations) subsets of atoms in the two structures as rigid clusters, and the differences between them as flexible regions. This technique is limited by the availability of NMR data or structures crystallized in multiple conformational states.

A more recent approach views the molecular bonds and interactions as rigid bars instead of springs and applies methods from *rigidity theory* to build the rigid cluster decomposition. The method gives immediate information on the molecule's flexibility: the presence of many small clusters in certain areas may be correlated with the small fluctuations responsible for the B-factors; the presence of a small number of *large* clusters may be indicative of a slow motion bringing these large clusters apart. Heuristics implementing rigidity-based decompositions of protein structures have been around for over 15 years, such as the stand-alone executable ProFlex-FIRST,^27^ the web server FlexWeb-FIRST,^19^ and, more recently, our own KinariWeb.^13^ Biologically relevant information has been demonstrated on a handful of protein structures^18,31,32^ without the need of detailed dynamic simulations. Yet, the model is not ready to provide convincing evidence that biologically relevant (structural and functional) information can be automatically extracted on a larger scale, or that it has predictive power.

### C. KINARI

In this paper, we present the second version of KINARI, an on-going computational project for **KIN**ematics **A**nd **RI**gidity Analysis of biomolecules, designed by the author and developed in her research group. KINARI is made available as the free open web server KinariWeb at http://kinari.cs.umass.edu. For comparison purposes, we refer to the first released version of the software^13^ as *Kinari-1* and use *Kinari-2* for the new version described here.

### D. Rigor and efficiency

The main attractiveness of the rigidity-theory-based approach comes from the computational speed of the proposed algorithms and from the mathematically robust formulation. Classical results from rigidity theory and an adaptation of efficient bipartite matching formulations led to a very fast combinatorial algorithm for bar-joint structures in two-dimensions, called the *pebble game.*^20^ A heuristic was subsequently proposed by Jacobs^32^ for three-dimensional structures and was applied to proteins. This generated a lot of interest in the mathematical community, leading to an advanced understanding of the theoretical underpinnings of the model and of the correctness and complexity analysis of several proposed algorithms for rigidity analysis of molecular structures. In particular, this new mathematical understanding led to the replacement of the original *bar-joint* protein model and of the associated pebble game heuristic used in FIRST^32^ (known to be correct in two- yet invalid in three-dimensions) with a body-bar-hinge model^13^ in KINARI. Results of Tay^35^ together with the recent proof of Katoh and Tanigawa^23^ for the *Molecular conjecture* guarantee both the validity of the model for a large class of molecular structures, and the correctness of the cluster decomposition produced by the corresponding pebble game family of algorithms developed in Ref. 29. This sets the modeling used in KINARI on a solid theoretical foundation.

*Kinari-1* was designed to analyze a single biomolecule at a time. It was tested and profiled in 2011 on approx. 28 000 entries from the Protein Data Bank (PDB); we know that it fails to run to completion on many other entries. This is due primarily to the idiosyncrasies of the PDB file formats and to the limitations in processing very large size molecules. The goal of *Kinari-2* is to overcome these limitations and to make possible the efficient *large scale* study of protein flexibility. Its newly redesigned web interface accepts an extended, diverse collection of biomolecules and molecular complexes. New efficient methods have been designed for processing large and very large structures, in particular, those with high degree of symmetry such as crystals and viruses.

### E. Validation and reproducibility

Coarse-grained models suffer from various shortcomings such as their dependency on modeling parameters. To make an analogy with experiments in the physical sciences, one may view each such model as an instrument with adjustable “knobs” for its parameters: these knobs must be fine tuned for the experiment to “catch the proper signal” and *validate* the model, i.e., reproduce a behavior observed by some other kind of instrument, either physical or software. In short, the lack of universally accepted values for the modeling parameters prevents them from having predictive power. For rigidity-based methods, studies^38^ have pointed out their sensitivity on cut-off values and other choices of parameters. One would hope that universal parameters, if they exist, should be inferable from large scale experiments. But a large scale (possibly open, community-based) validation effort requires full reproducibility of the computational experiments, and the availability of robust and reliable implementations. Yet, inconsistencies in the reported results obtained by different implementations of the rigidity-based method have been recently identified,^7^ triggering a need for scientific *reproducibility* of such computational experiments.

*Kinari-2* emphasizes the reproducibility of *in silico* experiments and makes substantially improved tools and biologically motivated applications available to the user. Its algorithms are re-designed and re-engineered to ensure efficient processing of large collections of *related* biomolecules, such as those obtained through simulated bond dilutions, mutations, conformational changes, or molecular simulations. The ultimate goal is to have KINARI succeed on a very high percentage of the data available in the PDB, the large publicly available repository of biomolecules,^2^ and, ultimately, to make possible a *large scale cross validation* of the rigidity analysis method and several other derivative applications.

## II. SINGLE-MOLECULE RIGIDITY ANALYSIS

**FIG. 1.**
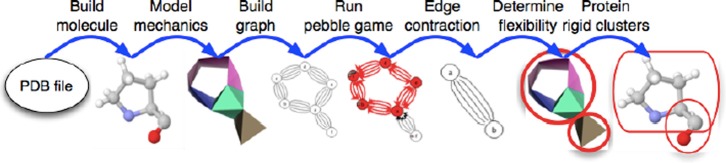
Steps in KINARI Rigidity analysis of the amino acid proline. The amino acid is first modeled as a mechanical bar-body-hinge network, which is used to build a multi-graph on which the pebble game is run. Based on the results of the pebble game, another bar-body-hinge framework is constructed and flexibility and rigid clusters are inferred from this framework. Adapted from Ref. 13.

**FIG. 2.**
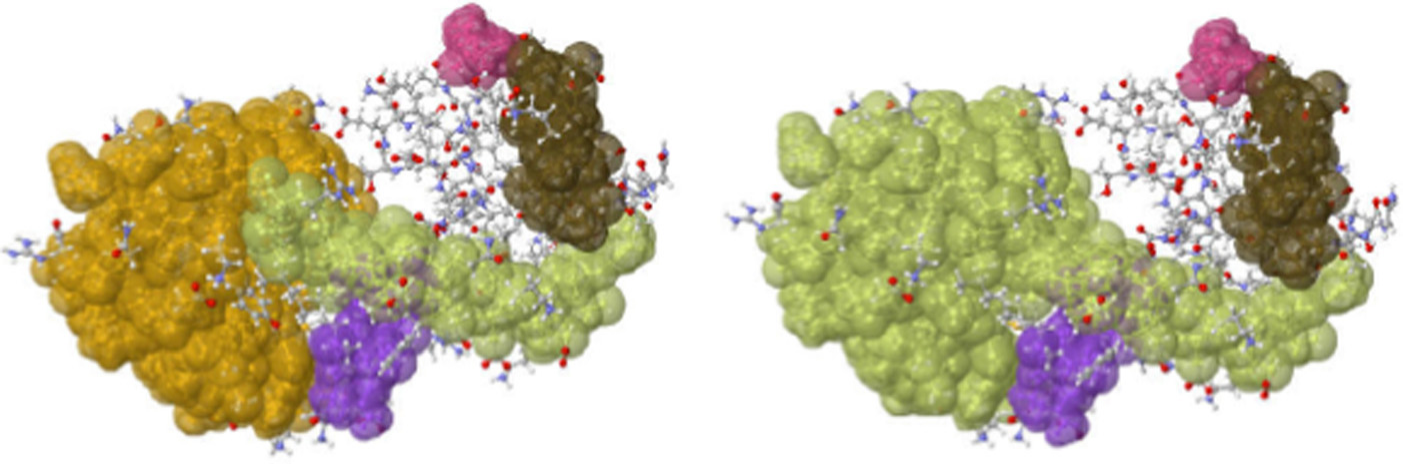
(Left) The rigid cluster decomposition for lysozyme 2LZM with standard modeling. (Right) The addition of two interactions joins two clusters into a larger one. Adapted from Ref. 13.

In KINARI, the rigidity analysis of a single molecule proceeds along the computational pipeline described in Fig. 1. The *input* is a PDB file. The *output* is a file describing the rigid cluster decomposition of the selected molecule. This file is subsequently sent to a JMol visualizer to produce interactive 3D images with colored clusters as in Fig. 2. An introduction to the method of rigidity analysis has been presented in Ref. 33 and is available on the KinariWeb site.

### A. Build the molecule

The first step is to extract from the input PDB file the molecules of interest and prepare them for the upcoming analysis. In *Kinari-1*, this pre-processing phase is called **Curation**: it attempts to generate *as-good-as-possible* data from the information found in the PDB file; this is in general noisy, and sometimes incomplete or presented in a non-standard manner. Unless ran with predefined default options, Curation is an interactive phase: the user may perform *in silico surgery* on the molecular complex present in the PDB file, by deleting solvent or ligands, selecting specific chains, or keeping an entire molecular complex. If bio-assembly or crystal information is available, the user may opt to work with such larger structures. Some necessary *repairs* are also performed, such as adding missing Hydrogen atoms (using the third-party software Reduce^39^) to structures solved by X-ray crystallography (they are needed for computing the hydrogen bonds). Finally, a network of various types of bonds and interactions between the atoms is computed. An energy value is calculated for each particular bond or interaction, according to its type, and the user specifies a cutoff value for hydrogen bonds and hydrophobic interactions. For details, we refer the reader to Ref. 13 and the on-line documentation available on the KinariWeb site.

It is important that the user understands the impact of the Curation phase on the rigidity analysis results, in particular, the choice of the energy cut-off value for hydrogen-bonds: this is one of the *tunable* parameters of the software “instrument.” Fig. 2 illustrates the strong impact that the addition of even a small number of such bonds can have on the results. Likewise, the presence or removal of ligands and other types of chains (such as those present in protein-nucleic acid complexes) can produce distinct rigid cluster decompositions.

### B. Mechanical model

This step is unique to KINARI, among existing rigidity analysis software implementations: from the previously computed collection of atoms and bonds, a mechanical body-bar-hinge structure is constructed, as follows. A body is assigned to an atom connected by covalent bonds to two or more other atoms, or to an entire peptide unit (without the residue); in short, a body corresponds to small groups of atoms known *a priori* to behave like *rigid units.* The bonds are then modeled as either hinges or as specific numbers of bars. Several other cases requiring special treatment are discussed in Refs. 12 and 14. By default, covalent and hydrogen bonds are modeled as hinges, and hydrophobic interactions as 3 bars, as these values appeared to produce rigidity results consistent with previous implementations and validated on experimental data. The user can experiment with other values of the bond modeling parameters, by using a number of bars between 1 and 6 for each type of chemical bond. This is the last step where the user can *tune the parameters* of the model.

### C. Build graph

Following a standard approach to analyzing body-bar-hinge frameworks, introduced in Ref. 34, this step builds a *multi-graph* by associating a node to each body, an edge to each bar and 5 edges to a hinge. This is the input to the main algorithmic procedure underlying rigidity analysis.

### D. Pebble game

An efficient, combinatorial algorithm referred to with the technical name of the *(6, 6)-pebble game* and described in full generality in Ref. 29 is run on the multi-graph built at the previous step. The algorithm is combinatorial as opposed to numeric: it does not depend on the atom coordinates or on numerical calculations such as the linear algebra required by GNM. The algorithm computes a decomposition of the multi-graph into *components*, which correspond to *rigid clusters* in the mechanical model. Unless the entire structure is rigid, the rigid clusters are connected (through hinges and bars) into a flexible framework. The mechanical interpretation is that any possible motion of the flexible framework maintains the rigid clusters, i.e., that the distances between any pair of points in a cluster are maintained. This code lies at the core of KINARI.

At the end of the pebble game algorithm, the result is converted from the multi-graph representation to a mechanical body-bar-hinge framework, with a cluster body comprising all the bodies in the corresponding rigid component and with the connections between these bodies being inherited from the initial body-bar-hinge model.

### E. Rigid clusters

Finally, everything is converted back to rigid clusters as collections of *atoms connected by bonds.* It is worth noticing that two such bodies may have in common two atoms connected by a bond acting as a hinge: this is not the case with the rigid clusters produced by FlexWeb-FIRST,^19^ which are disjoint.

### F. Visualization

*Kinari-1* provides an embedded JMol 3D visualizer for the rigid cluster decomposition of the biomolecule (Fig. 2). Typically, the user focuses on the large *dominant clusters*, which are colored according to size.

## III. BIG DATA IN *KINARI-2*

*Kinari-2* is far from being just a software refactoring of *Kinari-1*: the new design makes KINARI work on a large scale, on big data, in new biologically motivated applications and provides more user-friendly and intuitive tools. We describe now the specific software engineering, modeling, algorithmic, and mathematical problems that underlie this design.

### A. *Kinari-2* goals

#### 1. Data quality

The first, most basic and possibly the most tedious challenge we face is the *data quality bottleneck*: the large and often poorly documented variations in format and available information recorded (or not) in molecular structure files, and the difficulty of automatically extracting some of this information. To respond to the need for reproducible curation results, the new software tools (interactive or automated) should record the curator's decisions and generate quality measurements suitable for integration in further method-comparison or validation steps. In this category, we identified a need for going beyond existing tools for repairing molecular structures by developing or integrating third-party tools for handling missing parts (loops, residues, atoms), “fixing” low resolution protein structures or *C_α_*-only backbones, modeling and quantifying the impact of alternative conformations, detecting “defects” such as gaps in the protein structure (compared to the known protein sequence), or performing structural “repairs” in proteins. Not all the repairs needed by KINARI require a thorough structural validation, in the spirit of MolProbity.^8,9^ Rigidity analysis can proceed with less information, to which “guesses” (concerning potential interactions) can be added at any time (in an exploratory fashion) to evaluate their effect on the overall structural stability of the molecule.

#### 2. Scale

The second goal is to *scale* the existing algorithms to work on (almost) all the data from the reference database (PDB). For instance, *Kinari-1* was tested primarily on the asymmetric units of PDB files containing X-ray solved proteins and protein-nucleic acid complexes, but only on few biological assemblies. The challenges here arise from to size and time constraints in running the KINARI pipeline on very large molecular structures, as some of them may contain hundreds of thousands to millions of atoms. Indeed, some of the critical algorithms implemented in *Kinari-1* scale quadratically and require a revamping in logic and underlying data structures to work on such large molecules.

#### 3. Symmetries

One natural idea, which (to the best of our knowledge) was not explored until now in rigidity analysis software implementations, is to take advantage of existing *molecular symmetries* not just to build the large assembly but to avoid repeating calculations. In recent years, several fundamental mathematical and algorithmic problems concerning rigidity analysis for structures with symmetries have appeared in the literature, paving the way for algorithms with guaranteed theoretical properties and computational performance.

#### 4. Comparative validation

Various papers^16,31,32^ report on a number of studies on specific proteins, where the rigid cluster decomposition results obtained computationally with FIRST matched protein flexibility properties observed in lab experiments. However, more recently, slightly larger scale studies such as Refs. 17 and 38 observed that the method is sensitive (among others) to the placement of hydrogen bonds, and that there is no universal cut-off value for the hydrogen bond energy which would give biologically meaningful results for all the proteins in a specific dataset. These studies point to the need for systematic and comprehensive validation of rigidity analysis results in KINARI, and for building benchmarking datasets to assist with this goal.

Our approach is to compare the results obtained through our rigidity analysis with other coarse-grained implementations, in particular, on datasets for which validation studies have been conducted. These validation studies can themselves be by comparison with other approaches, but in the end the results of a cluster (or domain) decomposition should have been compared with biologically relevant properties of specific protein datasets. Moreover, we do not want just to make one series of runs on a system versus another and compare them. We want to provide a tool which is easy to extend for any kind of cluster-decomposition method and for any available dataset. Thus, from the very beginning we set as a goal a system in which all computational experiments would be fully reproducible and, in particular, conducted on similarly pre-processed or curated data.

#### 5. Large scale *in-silico* experiments

Efficient and robust rigidity and flexibility analysis of macromolecules may prove useful in various types of biologically motivated large scale *in silico* experiments. For instance, *hydrogen bond dilution* and *single point mutation* experiments have already been demonstrated to shed light on various aspects of functional properties of proteins.^30,31^ They may be useful in coarse-grained molecular simulations or may serve as brute-force filters prior to expensive *in vitro* mutagenesis experiments by providing useful information concerning the structural stability, or lack of it, for a mutated protein. Besides efficient software that carries out the calculations, there is a need for intuitive, visual tools to help the user interpret the results.

#### 6. Visualization

**FIG. 3.**
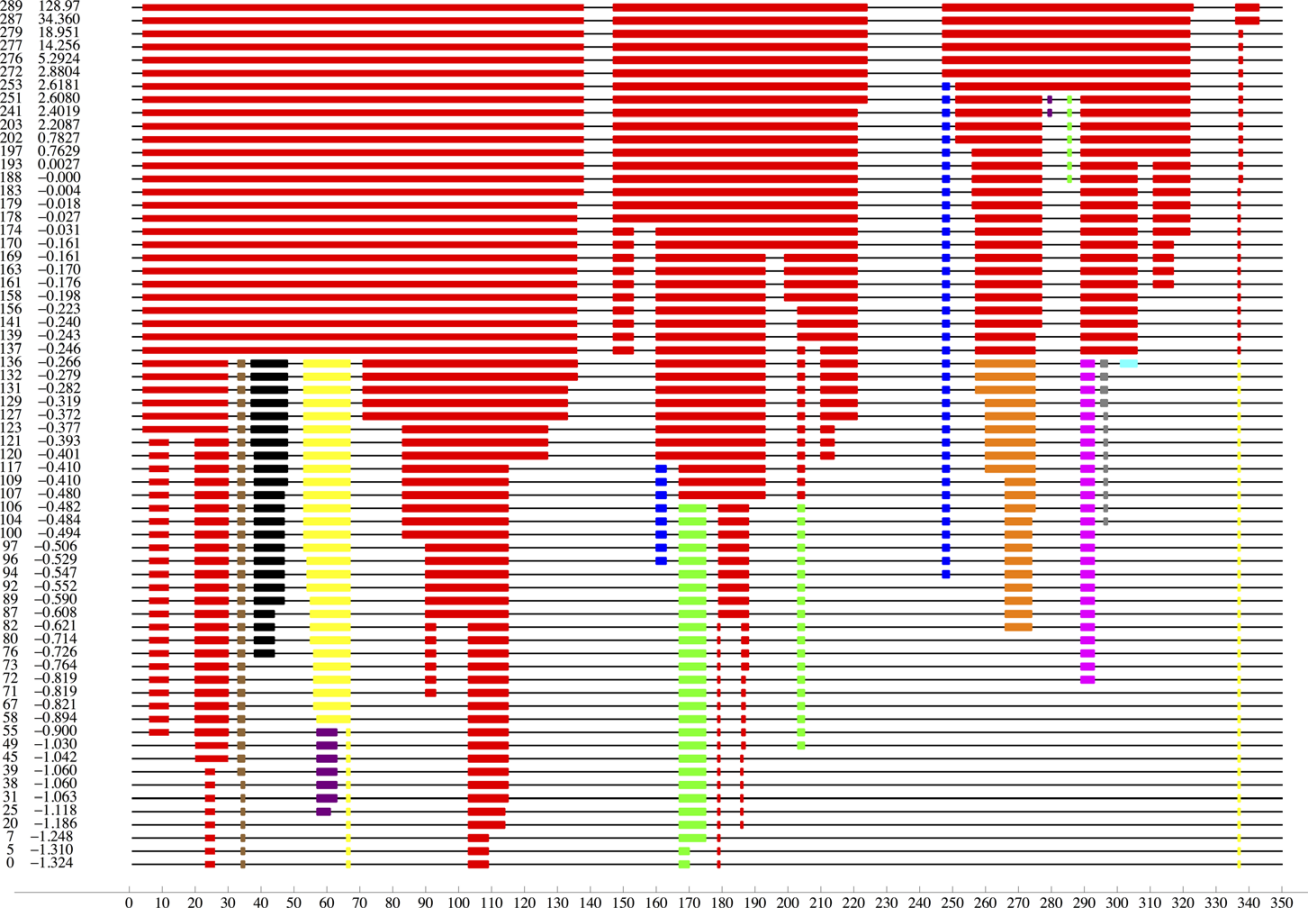
A 1D dilution plot for the analysis of rhodopsin 1L9H.

Finally, a major goal in our project is to develop *visualization* tools for interactive or automated comparisons of results from large scale rigidity analysis experiments of various kinds. This goes beyond the integration of JsMol to replace the previous Java-based JMol viewer. It requires algorithms and heuristics for *consistently coloring* cluster decompositions and domains obtained through different methods, on different proteins, on different models of the same protein, on different conformations of the same protein, etc. To illustrate the need, Fig. 3 shows the less intuitive one-dimensional dilution plot (as defined and used in Ref. 30) to visualize the outcome of a rigidity Dilution experiment.

**FIG. 4.**
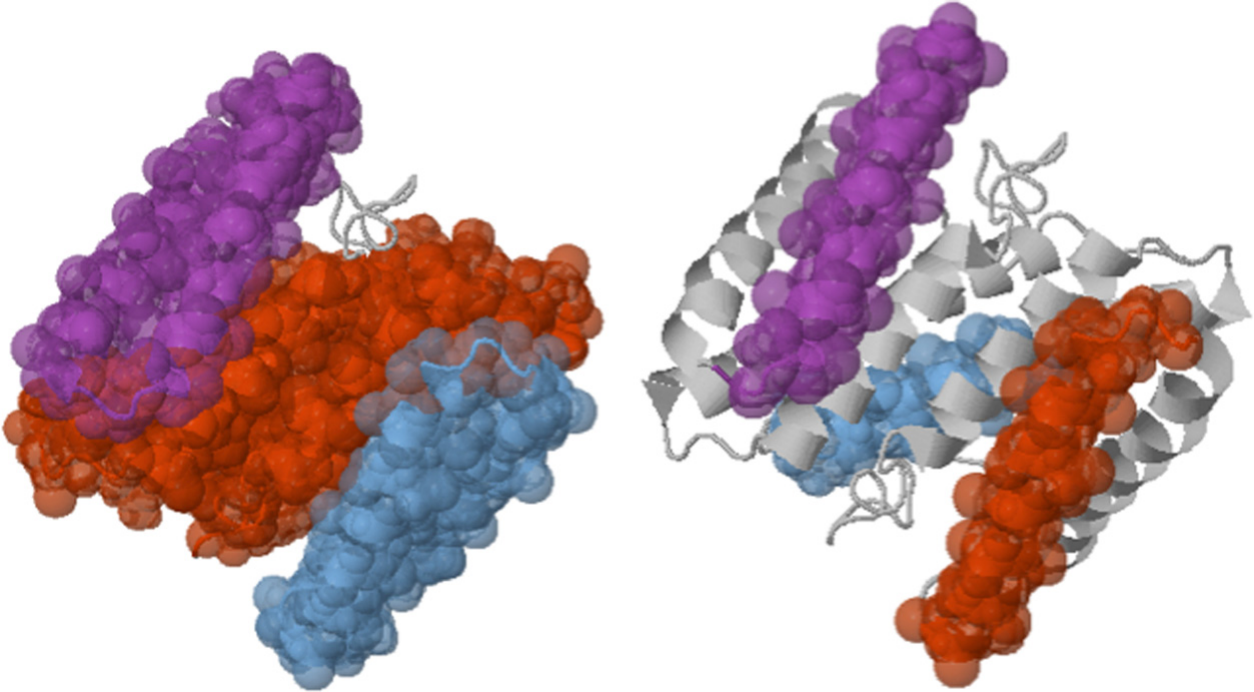
Two snapshots of a dilution experiment for 1BBH with *Kinari-1* coloring.

KINARI introduces a more suggestive, interactive, and integrated 3D visualization for the diluted clusters. However, a straightforward implementation in *Kinari-1* leads to the inconsistent coloring problem illustrated in Fig. 4 (for clarity, only the largest three rigid clusters are colored). In order to make it easy to compare visually two cluster decompositions of which one is a dilution of the other, it is desirable that atoms belonging to the largest subcluster of a previous cluster should inherit its color (this is not the case in Fig. 4, where colors are assigned by cluster size). The default coloring independently produced by *Kinari-1* at each stage of the dilution cannot guarantee this property (called *consistent coloring*). This problem is solved in *Kinari-2*. The prototype implementation of *consistent* 3D visualization for the two benchmark applications: *Kinari-Dilution* and *Kinari-Mutagen*, as well as further applications and ideas are presented in Refs. 10 and 11.

With these goals in mind, a complete redesign of the user support, file handling, navigation, and visualization front-end has been carried out and prototyped. A description of the system design decisions and front-end has appeared in Ref. 7. The system has a modular design and is ready for further upgrades integrating the specific or general solutions to the challenges described in Sec. III B.

### B. Challenges

#### 1. Advanced molecule building tools

The graph model on which rigidity analysis works is built in several stages, and only some of them make use of the geometric information present in the molecule's structure (atom coordinates): the addition of Hydrogen atoms and the calculation of weak bonds and interactions (hydrogen and disulphide bonds, hydrophobics, etc.). Therefore, we may attempt to partially reconstruct the strong bonds which are always present, and which do not require knowledge of the 3D structure, such as the standard bonds on the protein's or nucleic acid's backbone and in residues (bases), or standard hydrogen bonds and stacking interactions in double-stranded DNA. Similarly, once the bond network has been constructed, it can be edited by the user or by higher-level applications to add or remove interactions, and to model them with an appropriate number of edges prior to pebble game rigidity analysis. These coordinate-free molecule and model building tools can be used in prototyping and developing new interactive applications for molecular design using rigidity analysis results as guidelines.

***Advanced molecule building:***

***Develop methods for automated curation applicable to all the files in the PDB, in particular, for coordinate-free editing and repair.***

The main (and tedious) challenge here is to guarantee that all the cases that may be encountered in the PDB have been covered.

#### 2. Large structures with symmetry and periodicity

Symmetry and periodicity are pervasive in the PDB data: approx. 85% of its structures have been solved with X-ray crystallography. Many biomolecules deposited in the Protein Data Bank have symmetries and have to be analyzed in their *biological assembly* symmetric form rather than just the asymmetric unit. Furthermore, in Ref. 21 we have shown that the rigidity analysis of a crystallized protein may give qualitatively different rigid clusters when analyzed in isolation, without the neighboring cells, compared to an analysis that takes the crystal environment into account. Building small crystals increases substantially the size of the structure on which rigidity analysis is performed. The basic observation here is that some calculations are un-necessarily repeated when performed on structures with symmetry and/or periodicity: from building the molecule (curation) to building the mechanical model and the multi-graph, to running the pebble game and converting to rigid clusters.

***Rigidity of Large Symmetric Structures:***

***Develop algorithms to speed up rigidity analysis on biological assemblies and crystals, taking into account symmetries and periodicity.***

Naive extensions of the existing algorithms to account for symmetry and periodicity in rigidity theory may lead to mathematically incorrect results. The challenge is to build a robust mathematical theory for structures undergoing (or not) symmetrical or periodic deformations (and to understand the difference). This topic has recently received a lot of attention in rigidity theory and progress has been reported in a number of directions. In particular, we now have a thorough basis for understanding how *periodicity* affects rigidity analysis.^3–6^ Proper modifications of the pebble game algorithms to account correctly for periodic rigidity have been devised. They can be used as very efficient *alternative pre-calculations* for computing rigid clusters in crystals, since they work on the much smaller *quotient graph* of a periodic graph, rather than on the crystal fragment. The implementation in *Kinari-2* of these new algorithms, as well as devising heuristics in other cases that are not yet supported by a solid mathematical theory, is an on-going effort underlying two new KINARI apps: (a) rigidity analysis of viruses, with potential biological impact on better modeling and understanding viral assembly and (b) rigidity analysis of biological molecules in crystallized state, with potential biological impact on better modeling and understanding the flexibility of protein structures solved with X-ray crystallography.

#### 3. Large scale experiments for biological validation

Assuming that we have two methods to produce cluster decompositions of a biomolecule, we would like to run a comparison experiment on a large scale, tabulate and analyze the results: how efficiently can we do this, in a reproducible manner?

*Kinari-2* is designed to facilitate such survey-like experiments, which may include comparison of rigidity analysis with different modeling parameters, with different methods (such as GNM and KINARI), rigidity analysis of more complex (beyond single point) mutations of a single protein (which may produce huge datasets for an analysis that has many repeating parts across the dataset), etc. *Kinari-2* also provides tools for building curated datasets for carrying out such experiments in a reproducible manner. A more interesting challenge is to speed up such large scale experiments by taking advantage of similarities in the input data that could avoid un-necessary repetitions in the underlying calculations, beyond those identified through symmetries and periodicity.

***Large Scale Experiments:***

***Develop algorithms to speed up rigidity analysis on large datasets, possibly taking into account similarities in sequence, structure and the underlying mechanical model.***

#### 4. Visual comparison tools

**FIG. 5.**
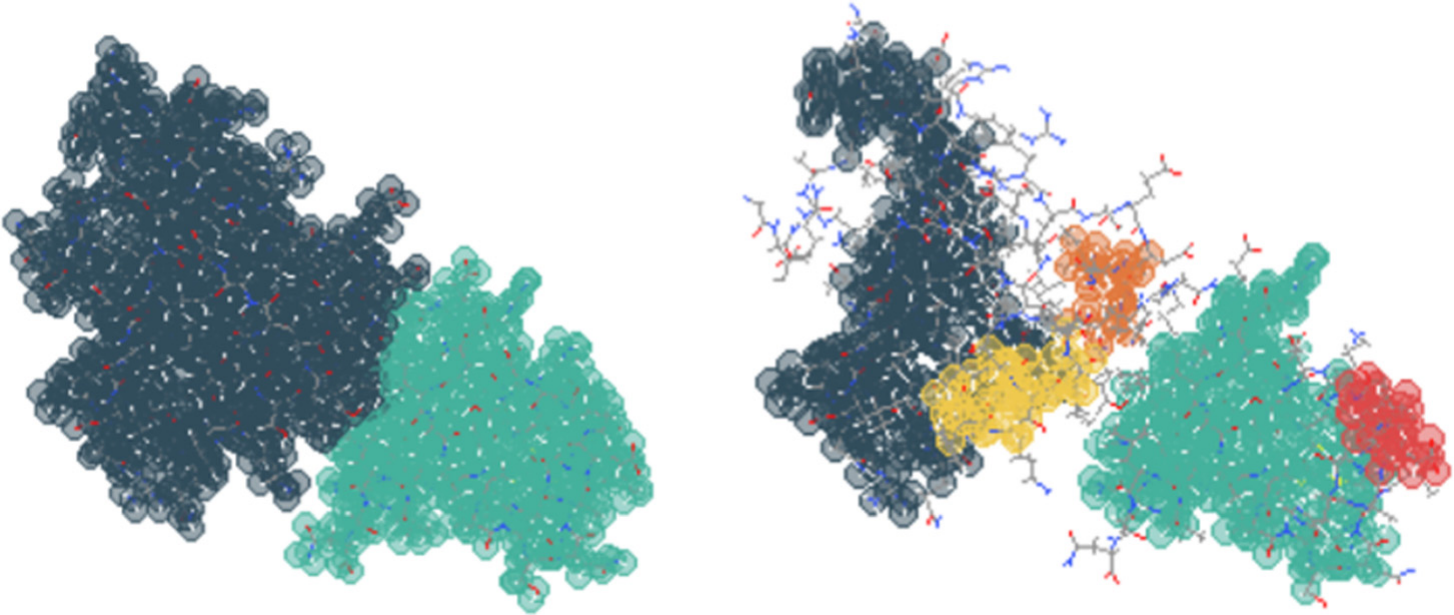
Comparative visualization of GNM and KINARI decompositions for chain B of 8ATC protein, with consistent coloring, shows a reasonably good correlation. The difference in cluster size comes from the fact that the GNM code of Ref. 28 consolidates GNM-computed clusters by adjoining to them smaller clusters within a certain distance. For proper comparison of methods, KINARI should apply a similar post-processing of its clusters.

We described above and illustrated in Fig. 4 one of the visualization problems arising in the Dilution application: since *Kinari-1* colors the clusters by size, inconsistent colorings may arise after several dilution steps. In a dilution experiment, hydrogen bonds are deleted in increasing order of their energy, hence new rigid clusters are obtained by the splitting of some previous cluster. A *consistent* coloring will have the largest of the new split clusters retain the color of the original cluster that was split. Satisfactory solutions have been developed for the *Kinari-2* applications of Dilution and Mutation analysis;^10,11^ the challenge is to automate the consistent coloring in more general cases, such as the one discussed below and illustrated in Fig. 5 (where the “good,” consistent coloring was produced manually).

***Consistent Domain Coloring:***

***Develop an automated method to consistently color rigid clusters for more general types of biologically motivated comparison applications.***

## IV. THE DESIGN OF *KINARI-2*

*Kinari-2* provides an infrastructure where *reproducible computational experiments* can be carried by all interested users using a web interface. Experiments are performed on single molecules or on datasets by running one of the apps provided in the current version. A collection of basic and advanced apps is provided, and the apps can be combined into more complex experiments. The basic apps handle the curation of a molecule (given as a PDB id or uploaded as a PDB-formated file by the user), building the mechanical model and the associated graph and running of the pebble game (with special handling of symmetries and periodicity) with calculation of the rigid clusters. The advanced apps include Dilution, Mutagenesis, Domain Comparison (currently providing comparison between GNM and KINARI), and others discussed below and compute consistent colorings for proper visualization of the results.

The server side application is implemented in PHP and Python and invokes JMol and binaries. It is hosted on an Apache web server. The user interface is written in HTML5, CSS, JavaScript, JQuery, and JsMol scripting. The specific infrastructure of the software system and the individual applications, experiments, and steps are described in Ref. 7. As highlights, we mention the use of the Model-View-Controller object-oriented software design paradigm, the availability of an intuitive navigation system through the steps of an application, tools for recording the relevant user-selected curation and modeling options and for managing the files produced by each experiment in a manner that ensures the reproducibility of the results.

### A. Reproducible experiments

#### 1. Users and experiments

*Kinari-2* introduces a system to manage users and their experiments in such a way that user privacy is guaranteed; it is easy to start and resume experiments, or transfer data from one experiment to another; the user can download all the files resulting from the computation done in *Kinari-2*, including a readable configuration file that keeps track of all the actions performed on the input PDB file; and the user can return, upload the previously saved files from some unfinished experiment, and resume the experiment. Besides this user and experiment management system, our new design has built-in capabilities for extending the system with new applications, which correspond to possible types of experiments. Each experiment consists in running one of these apps. A series of experiments can then be either manually or automatically streamed into a sequence, thus permitting the design of larger scale experiments on single molecules or on large datasets.

#### 2. Reproducibility of protein data curation

It is well known that the data deposited in the Protein Data Bank are not of uniform quality: to help with judging the quality of the experimental data deposited in the PDB, resolution and B-factors are parameters recorded with X-ray solved protein data. A number of entries in the PDB have been declared obsolete and replaced by others. Tools for checking the quality of crystallographic data are also available, such as MolProbity.^8,9^ The accuracy of the molecular model is relevant during the preprocessing of PDB files (prior to rigidity analysis) because of its implications on bond calculations. Bonds are computed with third party software, and different software performing the same task may produce different results, which may in turn lead to different rigidity analysis results. The critical steps include: adding the hydrogen atoms if the data come from an X-ray crystallography experiment; pruning the hydrogen bonds according to a user-selected cut-off value; selecting the model from among several available in a file containing data from an NMR experiment; computing the hydrogen bonds and hydrophobic interactions; building a biological assembly or, possibly, a small crystal, etc. Without precisely recording the entire sequence of steps performed during a molecule building (curation) experiment, the reproducibility of a subsequent rigidity analysis experiment may be compromised. Therefore, in *Kinari-2* we are placing maximum emphasis on the management and reproducibility of curation experiments.

### B. Building, editing, and repairing molecules and models

The Curation application starts by retrieving the file (from the PDB, from the user, or from a previously created dataset), after which a summary of the biomolecule is computed. This summary is displayed for the user to make an informed decision regarding the curation process. In the interactive version of the application, the software enters an *Editing* phase and proceeds along different branches depending on the molecular contents. The user is informed of the kind of files available for a given PDB code (asymmetric unit, bioassembly, or a bundle for a very large structure) and asked to select the desired option to be built. For NMR files, the user may select a specific model, or make a dataset of all existing models (for later performing rigidity analysis on all of them). For X-ray files, alternate configurations may be built. The user is also informed of the nature of the chains present in the file, and, if applicable, may choose to retain or prune the solvent, work only with proteins or include ligands, retain or prune other biomolecules such as DNA or RNA chains (if present), and select all or specific chains for the current experiment. This kind of detailed editing of the input file allows the user to carry out experiments to study the effect of a specific substrate or biomolecule on the flexibility or structural stability of a protein complex. The curation phase also works on datasets of related files, such as NMR ensembles or dynamic trajectories. In this case, the user may choose the same type of pre-processing, in order to study the effect of, say, a ligand or a specific chain present in each file.

Next after the *Editing* phase of curation, the user enters a *Repair* phase, where the selected molecular complex is processed for missing atoms (such as Hydrogens) or gaps along the backbone. The repairs carried out by *Kinari-2* may or may not contain structural information: some may be limited to connectivity information necessary for computing predictable, sequence-induced information, such as covalent bonds along the backbone and in the residues, but not hydrogen bonds or hydrophobic interactions. The user is informed of a predicted effect of the missing information on the rigidity analysis and will later have the option to experiment with conjectured interactions by adding them in a post-curation editing step.

A curated molecule is next processed to compute the set of all relevant bonds and interactions. They are recorded in separate categories, including covalent (single and double) bonds, peptide bonds, disulphide bonds, hydrogen bonds, and hydrophobic interactions. Scripts used in *Kinari-1* are adapted to be used in *Kinari-2*. For comparison purposes, alternate methods have been or will be integrated in *Kinari-2*, including bond calculations with third-party software such as JMol, HBPLUS, and bndlist.

Virus capsides make a separate group in the PDB and contain some of the largest deposited structures. Analyzing their rigidity (on full or partial assemblies) without taking into account the symmetries leads to prohibitively expensive calculations. On the other hand, a correct implementation that takes into account all of the symmetries is quite challenging and insufficiently investigated. For now, *Kinari-2* adopts a practical approach and builds a prototype to first test our ideas on the subset of icosahedral viruses, leaving more complex cases to be developed in future releases.

### C. Applications

Besides the basic apps for single-molecule curation and rigidity analysis, *Kinari-2* provides several advanced applications that either work on or generate large datasets for rigidity analysis. Some of them have been prototyped in *Kinari-1* by simply running the full pipeline on each structure of the dataset; this turns out to be very inefficient and prevents the further development of each application into a more accurate and responsive tool. In *Kinari-2*, efficiency is obtained by new algorithms and implementations for several application-specific versions of the kernel code (including the pebble game).

***Kinari-Mutagen***^22^ performs simulated point mutations to Alanine by removing corresponding hydrogen bonds and then runs rigidity analysis on all mutants. It ends with a comparative analysis for identifying the most significant mutations, which are likely to structurally destabilize the protein. In *Kinari-2*, this app relies on improved kernel algorithms for avoiding repetitive calculations and on a specific consistent coloring method for visualization.

***Kinari-ResidueSurgery*** is a step towards a more realistic version of Kinari-Mutagen. It performs simple checks for the *geometric feasibility* of the mutation of a residue *Res*1 to a residue *Res*2 and then performs rigidity analysis to evaluate the impact. It works by excising *Res*1 and then checking if *Res*2 fits, geometrically, in the space emptied by the excision; if so, it calculates potential bonds and interactions that may be formed. We remark that more realistic residue surgery applications will have to take into account conformational changes.

***Kinari-Dilution*** implements a simplified model for *protein unfolding* by removing one by one all the hydrogen bonds, in the order given by their calculated energy. Hydrogen-bond dilution is one of the first applications to demonstrate the usefulness of rigidity analysis and has been described in Refs. 30 and 31. Several subsequent protein dilution studies were conducted by other groups. With the existing tools provided by FIRST, the results are visualized and reported using a 1D comparison plot called a *dilution plot*, exemplified in Fig. 3 by its corresponding implementation in *Kinari-2*. The plot traces with similar colors the rigid clusters during dilution, along the protein sequence. In *Kinari-2*, we streamline the kernel code to avoid un-necessary re-runs of the pebble game, provide an improved 3D visualizer with a dilution-specific consistent colorings of the clusters, and offer several options for ordering the removed bonds in the dilution: besides the energy based version of Ref. 31, we provide geometrically induced orders (how deep a bond is inside the protein) or other criteria.

***Kinari-Redundancy***, described in Ref. 15 and developed originally as a prototype on top of *Kinari-1*, offers refined information about the stability of rigid clusters. A bond is *redundant* if its removal does not change the rigid cluster decomposition of the molecule, and *critical* otherwise; a cluster is redundant if it contains redundant bonds. Redundancy is a stronger indicator of structural stability than plain rigidity. In *Kinari-2*, this app is substantially improved with an efficient implementation of redundancy calculations in the new kernel and with consistent coloring when visualizing critical bonds.

***Kinari-Virus*** is a new application for rigidity experiments with assemblies and subassemblies of icosahedral viruses, leaving more complex cases to be developed in future releases. Its specialized visualizer allows for interactive exploration of symmetry-specific features in viruses.

***Kinari-Domains*** is a new application that allows for large scale comparison of cluster decompositions obtained by different methods. The current prototype implementation compares the GNM domain decomposition program^28^ with KINARI, but the underlying infrastructure is general and designed to be used for validation purposes. Fig. 5 illustrates one of these domain comparison experiments, from Ref. 36; it also points to one of the challenges encountered in automating such domain decomposition comparisons.

***Kinari-Conformations*** is a new application for rigidity experiments involving different conformations (or models) of the same structure. The conformations may come from PDB files containing NMR experimental data, alternative positions of atoms from X-ray solved structures, trajectory files from Molecular Dynamics simulations, etc. Its specialized visualizer allows for interactive exploration of rigid clusters across the family.

### D. Kernel modifications

#### 1. New features

The kernel of *Kinari-2* is being redesigned for faster, improved versions of current and new applications that necessitate repeated rigidity calculations on large datasets. Currently, we target applications where the datasets are comprised of (a) structurally related proteins, such as those obtained through simulated Dilutions, Mutations, or motion generating approaches, included Molecular Dynamics and GNM, and (b) simulated assemblies of structures with symmetries, such as viruses.

Several kernel structures and algorithms from *Kinari-1* need to be extended and adapted in *Kinari-2*, in order to handle efficiently structures with repetitions such as symmetry and/or periodicity, and to run efficiently on families of related structures such as diluted and mutated families and datasets of conformations of the same structure. This includes code for building the mechanical model and the multi-graph, as well as the pebble game.

As a preview, we illustrate the issues arising in the case of structures with periodicity. The mathematical terminology can be found in Ref. 3.

#### 2. Periodic rigidity kernel

**FIG. 6.**
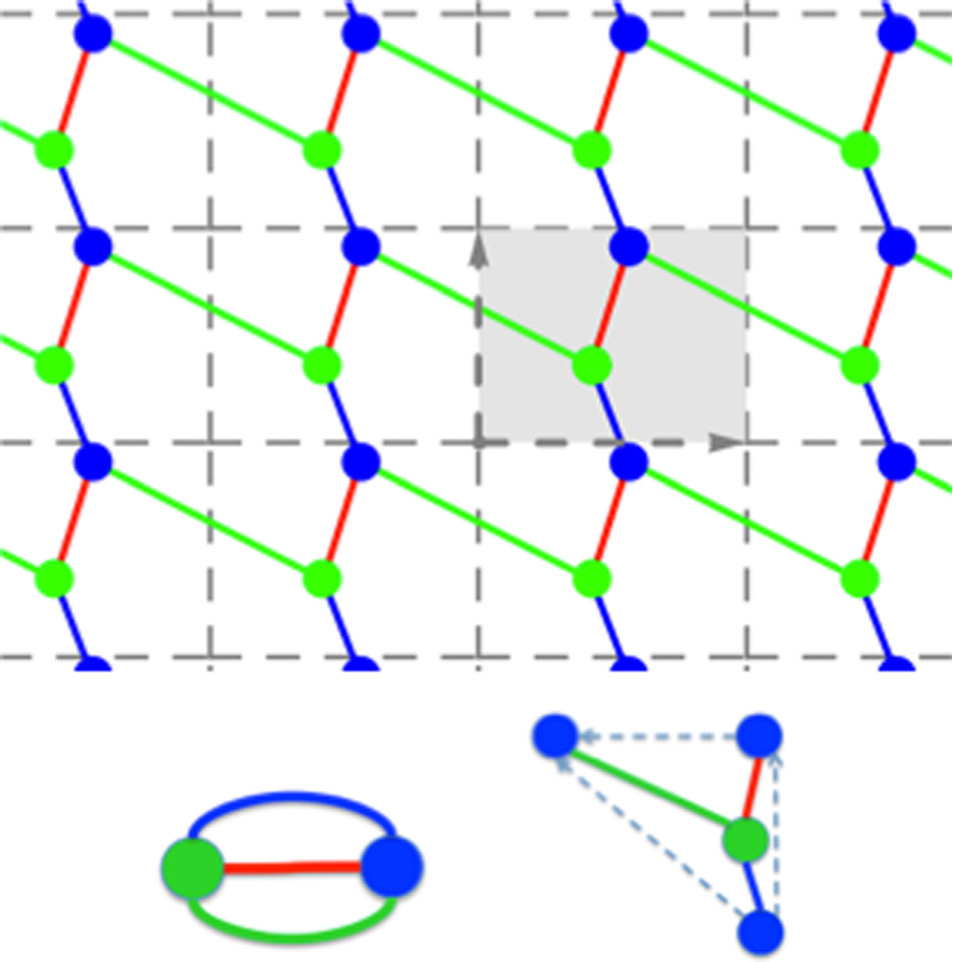
(Top) A 2D periodic graph, with unit cell and colored orbits of vertices and edges. (Bottom) The corresponding quotient graph, with matching vertex and edge orbit colors, and the schematic representation for vertex and edge representatives.

The most elaborate part is the calculation of the periodic structure (with unique representatives of atoms and bonds, resp. bodies, bars and hinges, resp. vertices and multi-edges) and corresponding quotient graphs. For this purpose, we designed new classes for internal data structures to store the periodic graph, the quotient graph, and the so-called schematic representation (illustrated in Fig. 6 for a 2D periodic graph). Two versions are prototyped for comparative results: one where we compute all the bonds and build the associated multi-graph for the large assembly prior to running rigidity analysis on it. The second version uses the quotient graph (based on either symmetries or periodicity) on which a slightly different algorithm will be run.

#### 3. Symmetric rigidity kernel

A complete treatment of symmetries extends the previous concepts in a more elaborate manner. In our implementation, we use an auxiliary connectivity graph whose nodes correspond to asymmetric units placed in the symmetric bioassembly, and edges denoting adjacency of specific units. Then we proceed with the calculation of the unique representatives of atoms and bonds, resp. bodies, bars and hinges, resp. vertices and multi-edges, and the corresponding quotient graphs. As in the periodic case, two versions are prototyped for comparative results: one where we compute all the bonds and build the associated multi-graph for the large assembly prior to running rigidity analysis on it. The second version uses the quotient graph (based on either symmetries or periodicity) on which a slightly different algorithm will be run.

#### 4. Repetitive rigidity kernel

For families of closely related structures, in particular, those obtained from Dilution and Mutation applications, the new *Kinari-2* kernel avoids repetitive calculations in an application-specific manner. A library is developed for these extended classes, with their associated data structures and methods: repetitive molecular structures, body-bar-hinge and multi-graph structures, and the corresponding pebble game algorithms.

### E. Application specific consistent coloring

*Kinari-1* implemented the core application of modeling, analyzing, and visualizing the rigidity of a *single model* of a *single molecule* in a single computational experiment. The goals of *Kinari-2* require more advanced *visual comparison tools*, capable of capturing essential common features of a variety of domain decompositions, on the same or on different molecules. These tools are designed to assist the user (a biologist) with a variety of tasks:

1. **Model fitting:** comparing the results of rigidity results for a protein analyzed with different mechanical modeling options (e.g., hydrogen bond bar-hinge modeling or cut-off value)
2. **Cross validation:** comparison of different decompositions (obtained through various computational methods, or assigned by a crystallographer) with each other and with KINARI rigid cluster decompositions
3. **Folding, unfolding, and reconfiguration processes:** (a) visualization of the rigidity of multiple NMR models of the same protein, (b) examination of the rigidity of multiple conformations of the same protein, and (c) simulation of the unfolding pathway by generating and analyzing the rigidity of multiple structures along a modelled denaturation process (**dilution analysis**)
4. **Mutagenesis:** comparison of cluster decompositions for *in silico* mutated structures with those of the original protein, to gain insights into potential destabilizing mutations.

**FIG. 7.**
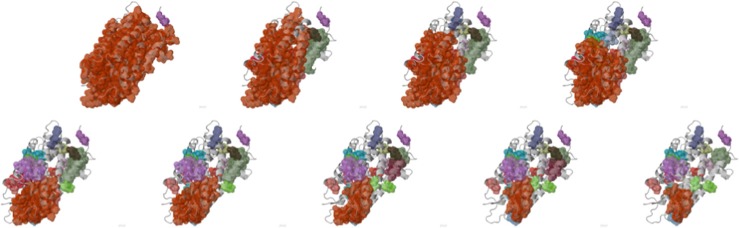
Consistently colored 3D Dilution Pathway of 1L9H chain A.

Visual comparison of rigidity and flexibility results is an essential part of this examination. Inconsistent coloring makes interpreting data from these biological comparisons difficult if not impossible. *Kinari-2* implements consistent coloring for the visualization of the complex apps. Once the computational experiment (Dilution, Mutation, Domain comparison, etc.) has been performed, an algorithm is run on the results to compute a consistent coloring. The algorithms underlying Dilution and Mutation colorings are simple. In the other cases, a heuristic based on the classical stable matching algorithm (described, e.g., in Ref. [24]) has been implemented; details appear in Ref. 10. Fig. 7 illustrates a consistently colored Dilution pathway, leading to advanced insights into what might constitute a *folding core*.

## V. CONCLUSION

We described the structure and design of *Kinari-2*, the second version of our freely available web server KinariWeb for protein rigidity and flexibility analysis. Its goal is to provide an integrated, open platform for reproducible *large scale* computational flexibility experiments on biomolecular data from the Protein Data Bank using techniques from rigidity theory. Our ultimate goal is to contribute to the biological cross-validation of various molecular flexibility methods by providing tools for large scale comparison of their results, and to develop *in silico* methodologies inspired by and serving the elucidation of biologically motivated problems. The system architecture presented here has been designed and prototyped to specifically handle the *big data* and *large scale* aspects of molecular flexibility problems. When fully integrated, the Kinari-2 system will be deployed at the same site as Kinari-1 as a freely available web server. Several other important challenges have been identified and remain to be addressed in future releases.
